# Recombinant α_1_-Microglobulin (rA1M) Protects against Hematopoietic and Renal Toxicity, Alone and in Combination with Amino Acids, in a ^177^Lu-DOTATATE Mouse Radiation Model

**DOI:** 10.3390/biom13060928

**Published:** 2023-06-01

**Authors:** Abdul Ghani Alattar, Amanda Kristiansson, Helena Karlsson, Suvi Vallius, Jonas Ahlstedt, Eva Forssell-Aronsson, Bo Åkerström, Sven-Erik Strand, Johan Flygare, Magnus Gram

**Affiliations:** 1 Division of Hematology and Transfusion Medicine, Department of Laboratory Medicine, Lund University, 221 84 Lund, Sweden; 2 Division of Molecular Medicine and Gene Therapy, Lund Stem Cell Center, Lund University, 221 84 Lund, Sweden; 3 Pediatrics, Department of Clinical Sciences Lund, Skåne University Hospital, Lund University, 221 84 Lund, Sweden; amanda.kristiansson@med.lu.se (A.K.);; 4 Department of Clinical Sciences Lund, CIPA, Lund University, 221 84 Lund, Sweden; 5 Department of Medical Radiation Sciences, Sahlgrenska Cancer Center, Institute of Clinical Sciences, Sahlgrenska Academy, University of Gothenburg, 413 45 Gothenburg, Sweden; 6 Department of Clinical Sciences Lund, Section for Infection Medicine, Lund University, 221 84 Lund, Sweden; 7 Department of Clinical Sciences Lund, Oncology, Lund University, 222 42 Lund, Sweden; 8 Department of Clinical Sciences Lund, Medical Radiation Physics, Lund University, 221 85 Lund, Sweden

**Keywords:** peptide receptor radionuclide therapy, α_1_-microglobulin, radiation, oxidative stress, bone marrow toxicity, renal damage, amino acids

## Abstract

^177^Lu-DOTATATE peptide receptor radionuclide therapy (PRRT) is used clinically to treat metastasized or unresectable neuroendocrine tumors (NETs). Although ^177^Lu-DOTATATE is mostly well tolerated in patients, bone marrow suppression and long-term renal toxicity are still side effects that should be considered. Amino acids are often used to minimize renal radiotoxicity, however, they are associated with nausea and vomiting in patients. α_1_-microglobulin (A1M) is an antioxidant with heme- and radical-scavenging abilities. A recombinant form (rA1M) has previously been shown to be renoprotective in preclinical models, including in PRRT-induced kidney damage. Here, we further investigated rA1M’s renal protective effect in a mouse ^177^Lu-DOTATATE model in terms of administration route and dosing regimen and as a combined therapy with amino acids (Vamin). Moreover, we investigated the protective effect of rA1M on peripheral blood and bone marrow cells, as well as circulatory biomarkers. Intravenous (i.v.) administration of rA1M reduced albuminuria levels and circulatory levels of the oxidative stress-related protein fibroblast growth factor-21 (FGF-21). Dual injections of rA1M (i.e., at 0 and 24 h post-^177^Lu-DOTATATE administration) preserved bone marrow cellularity and peripheral blood reticulocytes. Administration of Vamin, alone or in combination with rA1M, did not show any protection of bone marrow cellularity or peripheral reticulocytes. In conclusion, this study suggests that rA1M, administered i.v. for two consecutive days in conjunction with ^177^Lu-DOTATATE, may reduce hematopoietic and kidney toxicity during PRRT with ^177^Lu-DOTATATE.

## 1. Introduction

Neuroendocrine tumors (NETs) are a group of heterogenous tumors that can arise from different cells throughout the endocrine system, most commonly in the gastroenteropancreatic tract, lungs, and thymus [1,2]. Depending on the tumor site, type, and degree of spread, both the exhibited symptoms and overall survival vary significantly [3,4]. Peptide receptor radionuclide therapy (PRRT) is an established treatment to treat NETs that are metastasized or unresectable [5]. The treatment targets the overexpression of somatostatin receptors (SSTR), mainly SSTR type 2 (SSTR-2), on the cancer cells, and therefore has high uptake in somatostatin receptor imaging. By radiolabeling a somatostatin analog, radiation can be delivered specifically to cells where these radiopeptides are internalized after binding to SSTR [6]. Commonly used is the β-emitter lutetium (^177^Lu) coupled to the somatostatin analog (SSA) Tyr(3)-octreotate (TATE) through the chelator 1,4,7,10-tetraazacyclododecane-1,4,7,10-tetraacetic acid (DOTA), generally referred to as ^177^Lu-DOTATATE, which has high affinity for SSTR-2 [7]. Although tumor control is achieved in many patients, the current treatment regime (4 cycles of 7.4 GBq) is not sufficient for a complete cure, and the metastatic disease will progress in most patients [8,9].

In addition to tumor tissue, there is a rapid uptake of ^177^Lu-DOTATATE in certain healthy tissues, e.g., the kidneys, spleen, and liver, before it is excreted by the kidneys. Infusions of amino acids with positively charged side-groups are often used in PRRT and can help to reduce the renal tubular reabsorption of ^177^Lu-DOTATATE. Moreover, patients with reduced kidney function receive higher absorbed doses to the kidneys and bone marrow [10]. In addition, amino acids are often associated with nausea and vomiting, occurring in approximately half of the patients [8].

Even though PRRT with ^177^Lu-DOTATATE is generally well tolerated, bone marrow suppression occurs frequently in patients 4 to 6 weeks post-treatment [11]. Serious adverse events include myelodysplastic syndrome and acute leukemia with hematological toxicity (approx. 5–10% of grade 3 or 4) [12,13]. In the recent NETTER-1 trial, neutropenia, thrombocytopenia, and lymphopenia were reported in 1%, 2%, and 9%, respectively, of the patients (grade 3 or 4) with no renal failure during the median follow-up time (14 months) [8]. However, a recent systematic review reported an annual decrease of between 2 and 4 mL/min/1.73 m^2^ in glomerular filtration rates (GFR) following PRRT, suggesting that long-term renal toxicity might be a problem even with renal protection (i.e., amino acids, fractionated therapy, and limiting/individualizing activity) [14]. Therefore, despite several protective efforts, the kidneys and bone marrow remain the main dose-limiting organs due to sub-acute and/or long-term radiation toxicity. Hence, they remain an obstacle to overcome if increased, potentially curative, activities of radioactivity should be safely administered to patients.

α_1_-microglobulin (A1M) is a human antioxidant and reductase with radical-scavenging and heme-binding abilities. It is continuously produced in the liver, from where it is secreted into the bloodstream [15]. In a previous study, recombinant A1M (rA1M) protected kidney function and histological structure in a ^177^Lu-DOTATATE mouse model [16]. In addition, fewer animals died from radiation-induced damage. Importantly, rA1M has been shown not to interfere with tumor treatment or to protect cancer cells from the radiation damage [17]. More recently, rA1M was also shown to protect kidney functionality in a ^177^Lu-PSMA-617 radioligand therapy mouse model, further establishing the therapeutic effect of rA1M and the possibility of using it as a radioprotector [18].

In our previous study [16], the route of administration and dosage of rA1M was based on previous animal models [19,20,21]. However, optimal administration for PRRT might differ, thus, here we sought to compare different routes of administration and dosing of rA1M. In addition, recent data displayed a potential hemoprotective effect of rA1M [22,23], suggesting that protection against hematological toxicity, in addition to renal toxicity, might be a potential therapeutic target of rA1M. Therefore, hematological toxicity was investigated after injections of ^177^Lu-DOTATATE and rA1M. Lastly, amino acids can be used to ameliorate kidney damage during ^177^Lu-DOTATATE treatment in patients, and therefore we here investigated the combination of Vamin and rA1M to establish potential interference or synergy.

## 2. Materials and Methods

### 2.1. Recombinant Human A1M

Recombinant human A1M (rA1M, RMC-035, described in [19]) was provided by Guard Therapeutics International AB (Stockholm, Sweden).

### 2.2. Radiopharmaceuticals

Radiolabeling of the DOTATATE peptide with lutetium (^177^Lu, *E*_max_  =  0.5 MeV, half-life  =  6.73 days) chloride (LuMark, IDB, Baarle-Nassau, the Netherlands) was performed at Lund University Hospital (Lund, Sweden). Quality control of the resulting ^177^Lu-DOTATATE conjugate was performed at the Lund University Radionuclide Centre (Lund, Sweden).

Radiolabeling of A1M with ^125^I was done using the chloramine T method, as previously described [24]. Briefly, A1M and ^125^I (PerkinElmer, Waltham, MA, USA, NEZ033005MC) were mixed in 0.5 M sodium phosphate (pH 7.5) at final concentrations of 1 mg/mL and 10 mCi/mL, respectively. Chloramine T was added to 0.4 mg/mL and allowed to react on ice for 2 min, and the reaction was stopped by adding NaHSO_3_ to 0.8 mg/mL. Protein-bound iodine was separated from free iodide by gel-chromatography on a Sephadex G-25 column (PD10, GE Healthcare, Buckinghamshire, UK).

### 2.3. Animal Studies

All animal studies were performed with female BALB/cJBomTac mice (Taconic, Ejby, Denmark), which were 10 weeks old at arrival. Experiments were conducted in compliance with the national legislation on laboratory animals’ protection and with the approval of the regional Ethics Committee for Animal Research (Malmö/Lund, Sweden, permit numbers M5016, 15516/2017 and M12-16). Animals were kept in ventilated cages with food and water ad libitum.

### 2.4. Pharmacokinetics

For the pharmacokinetic study, mice were dosed subcutaneously (s.c., 20 mg/kg), intravenously (i.v., 5 mg/kg), and intraperitoneally (i.p., 20 mg/kg) (*n* = 8, for each group). Mice were conscious during sample collection and blood was sampled from the sublingual vein into lithium-heparin pre-coated vials (Microvette 100, Sarstedt, Germany). Three blood samples were collected from each of four mice in each group 5, 15-, 30-, 60-, and 180-min post-injection (s.c. and i.p.) and 1, 10-, 20-, 40-, and 80-min post-injection (i.v.). The concentration of injected rA1M in plasma was measured with an in-house sandwich ELISA. In brief, rA1M was captured by commercially available strips, pre-coated with anti-HIS-Tag antibodies (GenScript, Piscataway, NJ, USA). An rA1M calibrator and unknown samples were diluted in incubation buffer (10 mM Tris-HCl, 125 mM NaCl, 0.05% Tween-20, 2% BSA, pH 8.0). Then, the calibrator and unknown samples were incubated on the strips for 60 min (100 rpm at RT). The plate was washed three times (300 μL, 10 mM Tris-HCl, 125 mM NaCl, 0.05% Tween 20, pH 8.0). Next, HRP-labeled anti-A1M antibody (monoclonal 35.14, conc. 0.8 mg/mL, Innova Bioscience, Cambridge, UK) was added to each well and the plate was incubated for 60 min (100 rpm at RT). The plate was washed as above and TMB solution (SureBlue™ TMB 1-Component Microwell Peroxidase Substrate, KPL, Gaithersburg, MD, USA) was added. Thereafter, the plate was incubated in the dark until the reaction was stopped with 1 M sulfuric acid. The degree of binding was measured by absorbance at 450 nm using a VICTOR 1420 Multilabel Reader (Perkin-Elmer).

### 2.5. I.v. and S.c. Comparison

Animals received an i.v. activity of 150 MBq ^177^Lu-DOTATATE in conjunction with rA1M, administered either s.c. (20 mg/kg) or i.v. (5 mg/kg), or vehicle (10 mM Na-phosphate pH 7.4 + 0.15 M NaCl, 2 mg/mL histidine, in a volume corresponding to rA1M s.c. or i.v. administration). Control animals were administered i.v. NaCl (Sodium chloride, Braun 9 mg/mL, B. Braun Melsungen, Germany, in a volume corresponding to ^177^Lu-DOTATATE) and a vehicle (10 mM Na-phosphate pH 7.4 + 0.15 M NaCl, 2 mg/mL histidine, in a volume corresponding to s.c. or i.v. rA1M administration). Animals were sacrificed after 4 days. Before euthanasia, urine was collected and kept on dry ice. Blood for Luminex analysis was sampled from the vena cava of anesthetized animals (2–3% isoflurane, 0.2 L/m nitrous oxide, 0.2 L/m oxygen) and stored in lithium-heparin pre-coated vials (S-Monovette, Sarstedt, Germany).

### 2.6. Dose Escalation

Animals received an i.v. activity of 150 MBq ^177^Lu-DOTATATE (or corresponding volume of NaCl for control animals) with 1 mg/kg, 5 mg/kg, or 2 × 5 mg/kg (second dose given 24 h post-injection of ^177^Lu-DOTATATE) of rA1M (or corresponding volume of the vehicle, formulation as described in Section 2.5). Animals were sacrificed after 4 days. Before euthanasia, urine was collected and kept on dry ice. Peripheral blood for reticulocyte analysis was sampled from the vena saphena of non-anesthetized animals and stored in EDTA pre-coated vials (Microvette CB 300 K2E, Sarstedt, Germany). Bone marrow was harvested from the femur as described below.

### 2.7. Biodistribution

For the biodistribution study, eight mice were injected i.v. with ^125^I-labelled rA1M (5 mg/kg) and eight mice were injected i.v. with ^125^I-labelled A1M (5 mg/kg) and the positively charged amino acid solution, Vamin (Vamin 18N/l, Fresenius Kabi, Sweden, 35 mg/200 µL, administered i.p.). Four mice of each group were sacrificed after 10 min and the other four after 60 min. Kidneys, livers, spleens, and femurs were collected in scintillation tubes, weighed, and measured in a Nal (TI) well counter (Wallac Wizard 1480 Wizard, PerkinElmer). Results were corrected for background and radioactive decay. Organ specific uptake values were calculated as percent injected activity per gram of tissue (%IA/g).

### 2.8. rA1M and Vamin

Animals received an i.v. activity of 150 MBq ^177^Lu-DOTATATE (or NaCl for control animals, volume corresponding to that of DOTATATE) in conjunction with either the vehicle (volume corresponding to that of one dose of rA1M, formulation as described in Section 2.5), Vamin only (Vamin 18N/l, 35 mg/200 μL, administered i.p.), rA1M only (2 × 5 mg/kg, second dose given 24 h post-injection of ^177^Lu-DOTATATE), or a combination of Vamin and rA1M (2 × 5 mg/kg). Animals were sacrificed after 4 days. Before euthanasia, urine was collected and kept on dry ice. Blood for peripheral blood cell and reticulocyte counts was sampled from the vena saphena of non-anesthetized animals and stored in EDTA pre-coated vials (Microvette CB 300 K2E, Sarstedt). Bone marrow was harvested from the femur as described below.

### 2.9. Blood and Bone Marrow Analysis

Following the collection of peripheral blood, the reticulocyte percentage was determined using LSR Fortessa or Canto II flow cytometry using Retic-Count (BD Biosciences, San Jose, CA, USA).

Bone marrow cells were isolated by crushing femurs in phosphate buffered saline (pH 7.4) containing 2% fetalcalf serum (GIBCO, Waltham, MA, USA) and passing them though a 70 μm cell strainer to obtain single cell suspension. Bone marrow cellularity was counted from the single cell suspension using hematology analyzer SYSMEX KX-21N (Sysmex, Kobe, Japan).

### 2.10. Functional Urine Markers

Urine albumin was analyzed with the Mouse Albumin ELISA Kit (ab108792, Abcam, Cambridge, UK) according to the manufacturer’s instructions. The QuantiChrom™ Creatinine Assay Kit assay was performed according to the manufacturer’s instructions (DICT-500, BioAssay Systems, Hayward, CA, USA) to establish the albumin/creatinine ratio.

### 2.11. Plasma Markers

For detection of multiple mediators in plasma, Luminex Mouse Discovery Assay (8-Plex) (R&D Systems, Minneapolis, MA, USA) was used on a Luminex-xMAP/Bioplex-200 System with Bioplex Manager 5.0 software (Bio-Rad, Richmond, CA, USA).

### 2.12. Statistical Analysis

All statistical calculations were performed with GraphPad Prism (GraphPad Prism 9.4; GraphPad Software; GraphPad, Bethesda, MD, USA). Statistical tests are specified in the respective figure legends, and only significant differences are presented in the figures. Values of *p* < 0.05 were considered significant.

## 3. Results

### 3.1. Pharmacokinetics

**Figure 1:**
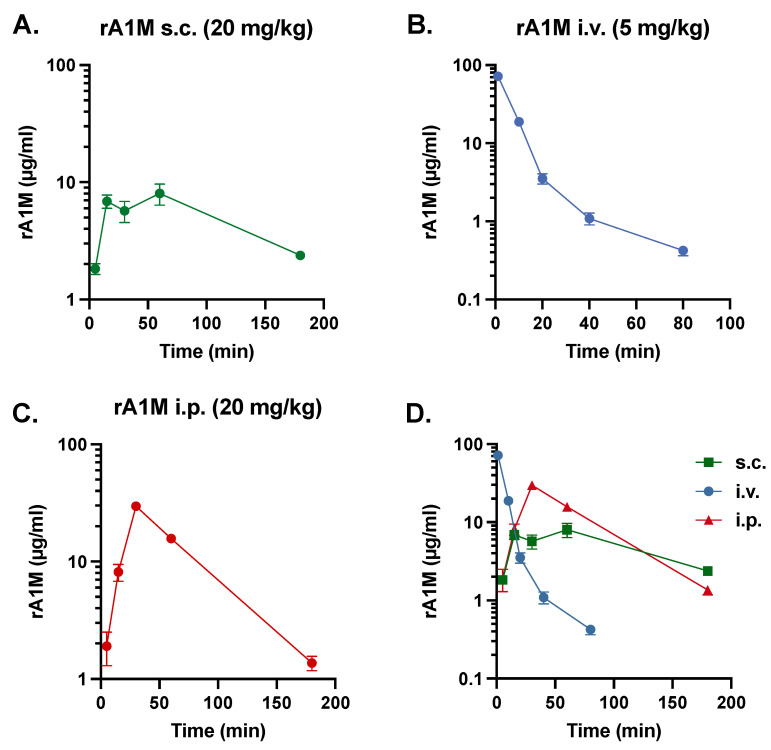
Pharmacokinetic study of rA1M. Plasma concentration of rA1M in female BALB/cJBomTac mice (*n* = 3–4/timepoint) after s.c. (20 mg/kg, (**A**)), i.v. (5 mg/kg, (**B**)), or i.p. (20 mg/kg, (**C**)) administration. In (**D**) all three administration routes are presented. Data is presented as mean ± SEM.

To investigate the pharmacokinetics of rA1M following different administration routes, animals were given s.c. (20 mg/kg), i.p. (20 mg/kg), or i.v. (5 mg/kg) injections of rA1M. The pharmacokinetic profiles are presented in Figure 1 and Table 1. As expected, s.c. administration had a slower transfer to plasma and a longer exposure time compared to i.v., whereas i.v. gave an initially higher concentration that rapidly decreased. In addition, i.p. administration of rA1M displayed a rapid increase and clearance from plasma. Moreover, administrating i.v. and i.p. resulted in a similar total exposure (AUC), although i.p. was administered in a four times higher dose. As a continuation, s.c., with a more even concentration over time, and i.v., with a direct and higher plasma concentration, were chosen for evaluation in the ^177^Lu-DOTATATE mouse model. In addition, these two routes would be most feasible for administration in humans.

### 3.2. Comparision of Protective Effects of S.c. vs. I.v. Administered rA1M

Earlier publications with rA1M in PRRT and radioligand therapy have shown a significant protective effect against kidney damage [16,18]. In our previous study we reported an absorbed dose of 42 ± 2.3 Gy to the kidneys (with 150 MBq of ^177^Lu-DOTATATE), comparable with the renal biological effective dose in clinical therapy where patients with good kidney and hematologic tolerance receive 40 ± 2 Gy [25]. Therefore, the equivalent activity was administered in the following experiments.

**Figure 2:**
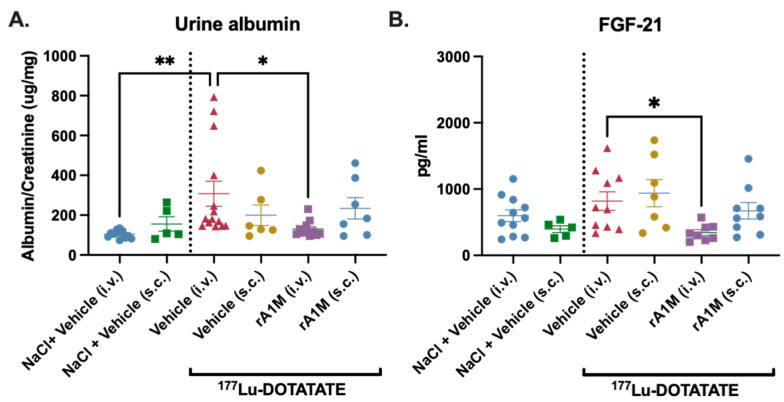
Evaluation of the protective effects of i.v. vs. s.c. administered rA1M. Albumin levels in urine (**A**) and plasma FGF-21 (**B**) were measured 4 days after dosing in animals receiving NaCl and the vehicle (i.v. or s.c., volume corresponding to that of 150 MBq ^177^Lu-DOTATATE or rA1M injections, respectively), 150 MBq ^177^Lu-DOTATATE with vehicle (i.v. or s.c., volume corresponding to that of rA1M injections), or with rA1M (i.v., 5 mg/kg or s.c., 20 mg/mg). Data is presented as scatter plots with mean ± SEM. Statistical comparison between groups was made with one-way ANOVA with a Šídák’s multiple comparisons post-hoc test. Comparison was made between groups with the same administration route and between rA1M i.v. and s.c. Only significant differences are presented in the figure. * *p* < 0.05, ** *p* < 0.01.

We evaluated the effects on proteinuria (albumin in urine corrected for creatinine) following s.c. and i.v. injections in mice receiving 150 MBq ^177^Lu-DOTATATE. In general, s.c. administration gave more dispersed results. Animals receiving rA1M i.v. had a significantly lower albumin/creatinine ratio and displayed a level more similar to control animals when compared with animals administered via the same route (Figure 2A). A general screen for biomarkers was performed with Luminex in plasma, and fibroblast growth factor (FGF) 21, a marker of oxidative stress, was shown to be affected by radiation. Interestingly, i.v. administered rA1M reduced FGF-21, whereas this was not observed following s.c. administration (Figure 2B).

Importantly, i.v. injections of rA1M gave more consistent and advantageous results, i.e., significant reduction in urine albumin and FGF-21 levels in connection with the need of a lower dose to obtain a protective effect (i.v., 5 mg/kg compared to s.c., 20 mg/kg). Therefore, i.v. administration of rA1M was chosen for continued optimization of the dosing regimen.

### 3.3. Dose Escalation of rA1M

**Figure 3:**
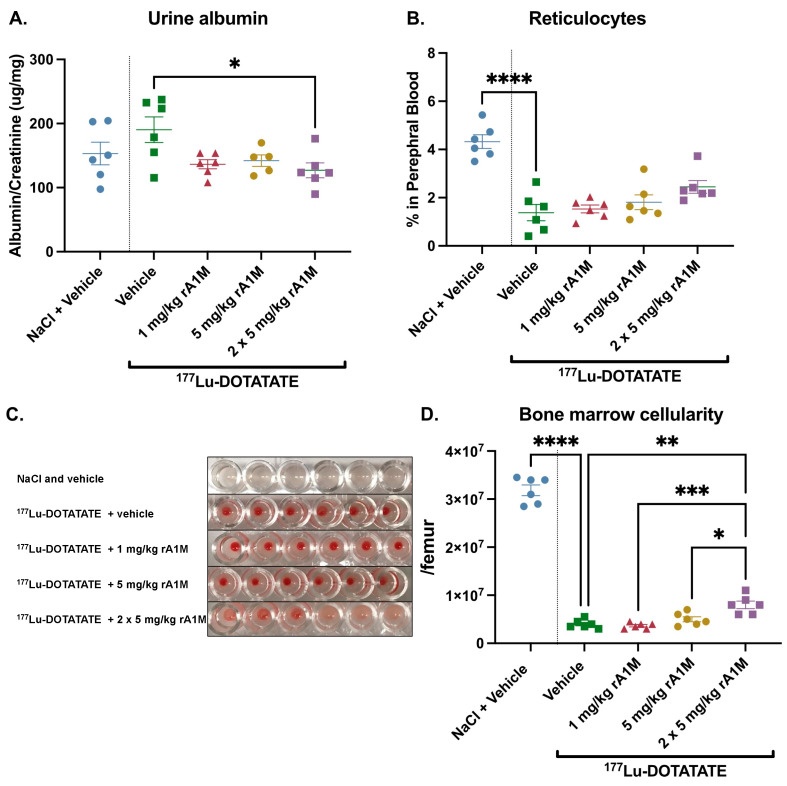
Evaluation of protective effect of different dosing regimen of rA1M. Urine albumin levels, corrected for creatinine (**A**), circulatory reticulocytes (**B**), representative image of bone marrow isolated single cells, showing red pellet as an effect of the irradiation treatment on depletion of white marrow (**C**), and bone marrow cellularity (**D**) were measured 4 days after dosing in animals receiving NaCl and the vehicle (volume corresponding to that of 150 MBq ^177^Lu-DOTATATE or one dose of rA1M injection), 150 MBq ^177^Lu-DOTATATE with vehicle (volume corresponding to that of one dose of rA1M injection), or with rA1M (1, 5 or 2 × 5 mg/kg). The representative gating strategies for flowcytometric evaluation are presented in Supplementary Figure S3. Data is presented as scatter plots with mean ± SEM. Statistical comparison between groups was made with one-way ANOVA with a Šídák’s multiple comparisons post hoc test. Comparison was made between ^177^Lu-DOTATATE + vehicle and all groups in addition to between the different doses of rA1M. Only significant differences are presented in the figure. * *p* < 0.05, ** *p* < 0.01, *** *p* < 0.001, **** *p* < 0.0001.

rA1M was administered i.v. in several different concentrations following ^177^Lu-DOTATATE injections (150 MBq): 1, 5 or 2 × 5 mg/kg. Administration of rA1M showed a clear trend towards reduced proteinuria (i.e., albumin/creatinine ratio) with all concentrations of rA1M, with 2 × 5 mg/kg resulting in a significant reduction (Figure 3A).

As described above, hematotoxicity, in addition to renal toxicity, constitutes one of the main limiting factors during ^177^Lu-DOTATATE therapy. In the current study, we evaluated the hematotoxic effects of ^177^Lu-DOTATATE (150 MBq) with or without rA1M (1, 5 or 2 × 5 mg/kg), focusing on bone marrow cellularity and reticulocyte percentage in peripheral blood. It was observed that the percentage of viable reticulocytes was significantly reduced in peripheral blood following exposure to ^177^Lu-DOTATATE. Administration of rA1M, prior to the ^177^Lu-DOTATATE administration, had an increasing trend of preserving reticulocyte population with increases in dose compared to the vehicle (Figure 3B), although this was not statistically significant. The cellular content of the bone marrow decreased drastically when mice were administered with ^177^Lu-DOTATATE. However, mice receiving rA1M showed a clear trend of preserving the white marrow, as observed by visualizing the total bone marrow cell pellet. The images show a clear preservation of the white marrow after two doses of rA1M (5 mg/kg/dose) compared to the vehicle group, as well as to the one rA1M dose (1 or 5 mg/kg) treated groups (Figure 3C, uncropped plate in Supplementary Figure S1). This was further confirmed by the total bone marrow cell count, displaying a significant protective effect of 2 × 5 mg/kg rA1M (Figure 3D), preserving twice the number of cells as ^177^Lu-DOTATATE alone.

**Figure 5:**
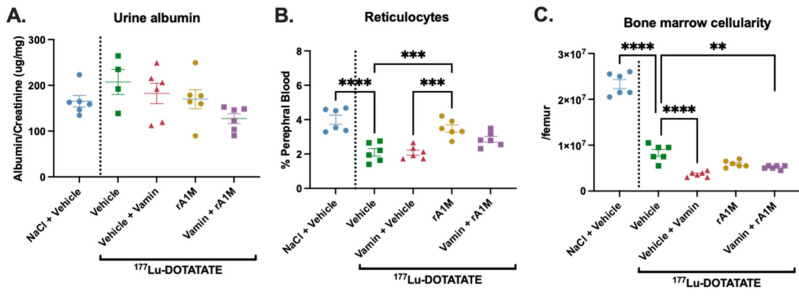
Impact of amino acid administration on the protective effects of rA1M. Urine albumin levels, corrected for creatinine (**A**), circulatory reticulocytes (**B**), and bone marrow cellularity (**C**) were measured 4 days after dosing in animals receiving NaCl and vehicle (volume corresponding to that of 150 MBq ^177^Lu-DOTATATE or rA1M injections, respectively), 150 MBq ^177^Lu-DOTATATE with the vehicle (volume corresponding to that of rA1M injections), the vehicle (volume corresponding to that of rA1M injections) and Vamin (35 mg/200 μL, administered i.p.), rA1M (2 × 5 mg/kg) or Vamin (35 mg/200 μL, administered i.p.), and rA1M (2 × 5 mg/kg). Data is presented as scatter plots with mean ± SEM. Statistical comparison between groups was made with one-way ANOVA with a Šídák’s multiple comparisons post hoc test. Comparison was made between ^177^Lu-DOTATATE + vehicle and all groups, and between ^177^Lu-DOTATATE + Vamin and rA1M with or without Vamin and between rA1M alone or rA1M + Vamin. Only significant differences are presented in the figure. ** *p* < 0.01, *** *p* < 0.001, **** *p* < 0.0001.

Additionally, by combining data from experiments presented in Figure 3 and Figure 5 (i.e., ^177^Lu-DOTATATE with or without 2 × 5 mg/kg rA1M), the reduction in albumin in urine as well as the preserved circulating reticulocytes in animals receiving rA1M were further emphasized (Supplementary Figure S2).

### 3.4. rA1M in Combination with Vamin

**Figure 4:**
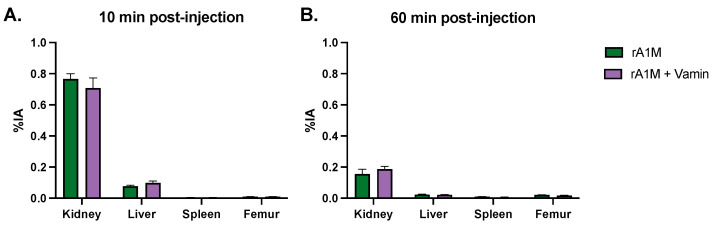
Impact of amino acid co-administration on rA1M biodistribution. Animals were injected with ^125^I-labelled rA1M alone (*n* = 8), or in combination with amino acids (Vamin, *n* = 8). Four (4) animals from each group were sacrificed after 10 (**A**) or 60 min (**B**). Organ specific uptake values were calculated as percent injected activity per gram tissue (%IA/g) in kidney, liver, spleen, and femur. Data is presented in bar graphs with mean ± SEM. No statistical differences between the two groups were detected following comparison between groups with a two-way ANOVA.

In the third part of this study, we investigated the possibility of combining infusion of amino acids with rA1M as a kidney protective measure. At first, the biodistribution of rA1M was investigated 10 and 60 min post-injection of rA1M alone or in combination with amino acids (Vamin, 35 mg/200 µL, administered i.p.). There was no significant difference in rA1M distribution to the organs investigated, i.e., kidney, liver, spleen, and femur, when combining with Vamin (Figure 4A,B and Supplementary Table S1).

Secondly, we examined albumin in urine (corrected for creatinine) following exposure to 150 MBq ^177^Lu-DOTATATE-injections alone, with Vamin (35 mg/200 µL, administered i.p.), with rA1M (2 × 5 mg/kg), or with a combination of rA1M (2 × 5 mg/kg) and Vamin (35 mg/200 µL, administered i.p.) (Figure 5A). Interestingly, the combination of rA1M and Vamin appears to have a synergistic effect and results in lower proteinuria levels compared to rA1M or Vamin alone, although this is not statistically significant. In peripheral blood, administration of rA1M, but not Vamin alone, significantly preserved the percentage of reticulocytes. The combination of Vamin and rA1M displayed a trend, although not significant, towards a preserved number of peripheral reticulocytes (Figure 5B). The cellular content of the bone marrow decreased when mice were exposed to 150 MBq ^177^Lu-DOTATATE, and mice receiving Vamin or rA1M + Vamin, had significantly fewer cells in the bone marrow (Figure 5C).

## 4. Discussion

In this work, we show that a recombinant version of the human reductase and heme- and radical-scavenger protein A1M may protect against hematopoietic and renal toxicity during ^177^Lu-DOTATATE radiation therapy. We also conclude that the biodistribution of rA1M is not significantly affected when amino acids are co-administered, suggesting that it can be feasible to administer them in combination to patients undergoing PRRT treatment.

The renal protective effect of rA1M during PRRT and, to a lesser extent, radioligand therapy with ^177^Lu-PSMA-617, have previously been described [16,18,26]. The preservation of renal function is believed to result from the molecular mechanisms of A1M, i.e., reductase activity and free radical scavenging, binding of free heme, and stabilization/preservation of mitochondrial function (reviewed in Åkerström et al., 2014; Kristiansson et al., 2021 [26,27]), in combination with its co-localization to the kidneys with ^177^Lu-DOTATATE after i.v. administration [24]. In an attempt to further optimize therapy, here, we investigated possible alternative administration routes of rA1M. Analyzing pharmacokinetics of rA1M after s.c., i.p., and i.v. administration, s.c. administration was observed to have a much lower plasma peak concentration (approximately 10%) than i.v. administration. Importantly, the renal protective effect in the mouse PRRT model, here evaluated by albuminuria, was only observed to be significant following i.v. administration. Although this could indicate a difference in the mechanism of action or efficacy of rA1M depending on the administration route, it is our conception that the level and/or timing of the peak rA1M concentration constitute important aspects in the renal protection during radiation therapy. In fact, this is further supported by a previous study by Kristiansson et al., where 7 mg/kg of rA1M showed a protective effect in the ^177^Lu-DOTATATE model [16]. Thus, it could be that s.c. administration would require earlier administration in order to have its peak concentration correlate with the peak concentration of ^177^Lu-DOTATATE in the kidneys, or that an even higher dose (i.e., 20 mg/kg) would be needed to confer the same protective effect as an i.v. administration of 5 mg/kg.

In addition to the administration route, we evaluated different dosing regimens of rA1M, i.e., two doses (1 and 5 mg/kg) and repeated dosing (2 × 5 mg/kg). When a second dose of rA1M was administered 24 h post-injection of ^177^Lu-DOTATATE, the protective effect on kidneys, bone marrow cellularity, and reticulocytes were improved. One can speculate that the first dose of rA1M targets the immediate effect of the radiation (i.e., protects against direct DNA-damage and reactive oxygen species resulting from radiolysis), whereas the second dose reduces secondary damage (i.e., lesions on cell and extracellular matrix components) and oxidative stress resulting from cell debris, etc., from cells dying following exposure to the radiation.

Today, mostly mild reversible hematological toxicity is seen in patients after PRRT. However, in advanced cases with high tumor burden, bone metastasis, or when patients have been pretreated with alkylating-based chemotherapy or myelotoxic therapies, patients have a higher risk of long-term toxicity including myelodysplastic syndrome (MDS) and acute myeloid leukemia (AML) [28,29]. Moreover, patients with decreased kidney function also have an increased risk of bone marrow toxicity, and women are reported to be more likely to suffer from thrombocytopenia and anemia after PRRT than men [10,30]. Here, we report for the first time that rA1M may decrease hematopoietic toxicity, particularly preserving reticulocytes during ^177^Lu-DOTATATE radiation therapy. The protective effect observed on erythropoiesis can possibly be linked to the ability to mitigate intracellular heme toxicity. It is possible that radiation induces a block in the cap-dependent translation of globin mRNA [31] and leads to heme accumulation, analogous to ribosome deficiency causing erythroblast heme toxicity in Diamond–Blackfan anemia [32]. In addition, rA1M may have a more general, cell- and membrane-stabilizing effect on circulating cells, consistent with previous data indicating a role in RBC protection [22,23]. However, the radical scavenging and antioxidative properties of rA1M, resulting in elimination of oxidative stress, might be the main mechanisms also behind the reduced cell death in the bone marrow [33].

FGF-21 expression increases in response to mitochondrial disease, oxidative stress, and physical stress, resulting in elevated plasma levels [34]. Moreover, FGF-21 has been shown to increase in numerous diseases, e.g., nephropathy, where levels correlate with plasma, blood urea nitrogen, and creatinine as well as proteinuria [35]. Here, we observed a similar pattern: groups with higher levels of albumin in urine also displayed higher levels of plasma FGF-21. Treatment with rA1M was effective when administered i.v., resulting in significantly lowered FGF-21 plasma levels. Since s.c. administration of rA1M did not yield significant renal protection nor FGF-21 reduction, this could imply that the effect on FGF-21 is related to renal protection. However, since rA1M, in addition, is an antioxidant and a heme- and radical-scavenger that has been shown to have a mitochondrial protective effect [36,37], it is difficult to attribute this only to kidney protection. Hence, the lower FGF-21 levels in rA1M treated animals could be attributed to a general lowering of the widespread physical stress resulting from the ^177^Lu-DOTATATE radiation exposure.

Amino acids are used in clinical practice to reduce renal side effects during PRRT. If rA1M is to be included in the treatment, it might be used alone or concurrently with amino acids. In the current study, we therefore investigated the use of rA1M and amino acids (Vamin), alone or in combination. First, we investigated the biodistribution of rA1M together with Vamin, which indicated that Vamin would not alter the biodistribution of rA1M. Interestingly, the renal uptake was not affected with Vamin, which might have been expected considering that A1M is taken up by proximal tubular reabsorption. The mechanism behind this remains unclear and needs further investigation. Next, we evaluated the effects of rA1M and Vamin, alone or in combination, in the ^177^Lu-DOTATATE radiation mouse model. The combination of Vamin and rA1M had a synergistic effect and had a trend towards lowered renal damage (measured here as albuminuria), although this was not statistically significant. This enhanced effect of combining rA1M and Vamin might be the result of amino acids reducing the renal uptake of ^177^Lu-DOTATATE by ~50% by competing with the megalin/cubilin complex. Although this otherwise results in retainment of the radiopeptides in the renal interstitium [38], rA1Ms co-localization with ^177^Lu-DOTATATE most probably reduces the resulting oxidative cascade from the radiation.

Interestingly, no protective effect was seen on bone marrow cellularity or circulating peripheral blood reticulocytes with Vamin alone; instead, the addition of Vamin seemed to reduce the effect of rA1M. The molecular mechanism behind this effect is unclear and will require additional future studies to be better understood.

rA1M was recently investigated in a Phase 1b clinical study in cardiac surgery patients, a procedure often associated with acute kidney injury, and was shown to be well tolerated with plasma exposures in the expected pharmacological activity range. In addition to safe administration, results also suggested that rA1M reduced perioperative kidney cell damage [39]. Although additional research is needed, this further supports the potential use of rA1M as a kidney protector in various clinical conditions.

In conclusion, our data strengthen previous published data suggesting the use of rA1M as a radioprotector during PRRT [16,17]. We show that combining rA1M with amino acids might be possible, and that in addition to renal protection, rA1M may reduce hematopoietic toxicity.

## Figures and Tables

**Table 1:** Route of administration of rA1M, dose, mean AUC with 95% confidence interval, and t_max_.

Group	Route of Administration	Dose	AUC (95% CI)	t_(max)_
rA1M s.c.	s.c.	20 mg/kg	966.2 (559.1–1373)	60 min
rA1M i.v.	i.v.	5 mg/kg	595.1 (458.7–731.6)	1 min
rA1M i.p.	i.p.	20 mg/kg	2040 (1809–2272)	30 min

## Data Availability

Data available on request.

## Supplementary Materials

The following supporting information can be downloaded at: https://www.mdpi.com/article/10.3390/biom13060928/s1, Figure S1: Preservation of white marrow.; Figure S2: The protective effects of rA1M.; Figure S3: The representative gating strategies for flowcytometric evaluation.; Supplementary Table S1: rA1M biodistribution with or without Vamin.

## References

[1] Kulke M.H., Shah M.H., Benson A.B., Bergsland E., Berlin J.D., Blaszkowsky L.S., Emerson L., Engstrom P.F., Fanta P., Giordano T. (2015). Neuroendocrine tumors, version 1.2015. J. Natl. Compr. Cancer Netw..

[2] Oronsky B., Ma P.C., Morgensztern D., Carter C.A. (2017). Nothing But NET: A Review of Neuroendocrine Tumors and Carcinomas. Neoplasia.

[3] Kaupp-Roberts S., Srirajaskanthan R., Ramage J.K. (2015). Symptoms and quality of life in gastroenteropancreatic neuroendocrine tumours. EMJ Oncol..

[4] Dasari A., Shen C., Halperin D., Zhao B., Zhou S., Xu Y., Shih T., Yao J.C. (2017). Trends in the Incidence, Prevalence, and Survival Outcomes in Patients with Neuroendocrine Tumors in the United States. JAMA Oncol..

[5] Camus B., Cottereau A.S., Palmieri L.J., Dermine S., Tenenbaum F., Brezault C., Coriat R. (2021). Indications of Peptide Receptor Radionuclide Therapy (PRRT) in Gastroenteropancreatic and Pulmonary Neuroendocrine Tumors: An Updated Review. J. Clin. Med..

[6] Sorbye H., Kong G., Grozinsky-Glasberg S. (2020). PRRT in high-grade gastroenteropancreatic neuroendocrine neoplasms (WHO G3). Endocr.-Relat. Cancer.

[7] Reubi J.C., Schär J.-C., Waser B., Wenger S., Heppeler A., Schmitt J.S., Mäcke H.R. (2000). Affinity profiles for human somatostatin receptor subtypes SST1–SST5 of somatostatin radiotracers selected for scintigraphic and radiotherapeutic use. Eur. J. Nucl. Med..

[8] Strosberg J., El-Haddad G., Wolin E., Hendifar A., Yao J., Chasen B., Mittra E., Kunz P.L., Kulke M.H., Jacene H. (2017). Phase 3 Trial of 177Lu-Dotatate for Midgut Neuroendocrine Tumors. N. Engl. J. Med..

[9] Kwekkeboom D.J., de Herder W.W., Kam B.L., van Eijck C.H., van Essen M., Kooij P.P., Feelders R.A., van Aken M.O., Krenning E.P. (2008). Treatment with the radiolabeled somatostatin analog [177Lu-DOTA0, Tyr3] octreotate: Toxicity, efficacy, and survival. J. Clin. Oncol..

[10] Svensson J., Berg G., Wängberg B., Larsson M., Forssell-Aronsson E., Bernhardt P. (2015). Renal function affects absorbed dose to the kidneys and haematological toxicity during 177Lu-DOTATATE treatment. Eur. J. Nucl. Med. Mol. Imaging.

[11] Brabander T., Teunissen J.J.M., Van Eijck C.H.J., Franssen G.J.H., Feelders R.A., de Herder W.W., Kwekkeboom D.J. (2016). Peptide receptor radionuclide therapy of neuroendocrine tumours. Best Pract. Res. Clin. Endocrinol. Metab..

[12] Bergsma H., Konijnenberg M.W., Kam B.L., Teunissen J.J., Kooij P.P., de Herder W.W., Franssen G.J., van Eijck C.H., Krenning E.P., Kwekkeboom D.J. (2016). Subacute haematotoxicity after PRRT with (177)Lu-DOTA-octreotate: Prognostic factors, incidence and course. Eur. J. Nucl. Med. Mol. Imaging.

[13] Bergsma H., van Lom K., Raaijmakers M.H., Konijnenberg M., Kam B.B.L., Teunissen J.J., de Herder W.W., Krenning E.P., Kwekkeboom D.J. (2018). Persistent hematologic dysfunction after peptide receptor radionuclide therapy with 177Lu-DOTATATE: Incidence, course, and predicting factors in patients with gastroenteropancreatic neuroendocrine tumors. J. Nucl. Med..

[14] Stolniceanu C.R., Nistor I., Bilha S.C., Constantin V., Simona V., Matovic M., Stefanescu C., Covic A. (2020). Nephrotoxicity/renal failure after therapy with 90Yttrium- and 177Lutetium-radiolabeled somatostatin analogs in different types of neuroendocrine tumors: A systematic review. Nucl. Med. Commun..

[15] Bergwik J., Kristiansson A., Allhorn M., Gram M., Åkerström B. (2021). Structure, Functions, and Physiological Roles of the Lipocalin α1-Microglobulin (A1M). Front. Physiol..

[16] Kristiansson A., Ahlstedt J., Holmqvist B., Brinte A., Tran T.A., Forssell-Aronsson E., Strand S.E., Gram M., Åkerström B. (2019). Protection of Kidney Function with Human Antioxidation Protein alpha1-Microglobulin in a Mouse (177)Lu-DOTATATE Radiation Therapy Model. Antioxid. Redox Signal..

[17] Andersson C.K., Shubbar E., Schüler E., Åkerström B., Gram M., Forssell-Aronsson E.B. (2019). Recombinant α1-Microglobulin Is a Potential Kidney Protector in 177Lu-Octreotate Treatment of Neuroendocrine Tumors. J. Nucl. Med..

[18] Kristiansson A., Örbom A., Ahlstedt J., Karlsson H., Zedan W., Gram M., Åkerström B., Strand S.E., Altai M., Strand J. (2021). (177)Lu-PSMA-617 Therapy in Mice, with or without the Antioxidant α(1)-Microglobulin (A1M), Including Kidney Damage Assessment Using (99m)Tc-MAG3 Imaging. Biomolecules.

[19] Åkerström B., Rosenlöf L., Hägerwall A., Rutardottir S., Ahlstedt J., Johansson M.E., Erlandsson L., Allhorn M., Gram M. (2019). rA1M-035, a Physicochemically Improved Human Recombinant α(1)-Microglobulin, Has Therapeutic Effects in Rhabdomyolysis-Induced Acute Kidney Injury. Antioxid. Redox Signal..

[20] Nääv Å., Erlandsson L., Axelsson J., Larsson I., Johansson M., Wester-Rosenlöf L., Mörgelin M., Casslén V., Gram M., Åkerström B. (2015). A1M Ameliorates Preeclampsia-Like Symptoms in Placenta and Kidney Induced by Cell-Free Fetal Hemoglobin in Rabbit. PLoS ONE.

[21] Wester-Rosenlöf L., Casslén V., Axelsson J., Edström-Hägerwall A., Gram M., Holmqvist M., Johansson M.E., Larsson I., Ley D., Marsal K. (2014). A1M/α1-Microglobulin Protects from Heme-Induced Placental and Renal Damage in a Pregnant Sheep Model of Preeclampsia. PLoS ONE.

[22] Kristiansson A., Gram M., Flygare J., Hansson S.R., Åkerström B., Storry J.R. (2020). The Role of α1-Microglobulin (A1M) in Erythropoiesis and Erythrocyte Homeostasis—Therapeutic Opportunities in Hemolytic Conditions. Int. J. Mol. Sci..

[23] Kristiansson A., Bergwik J., Alattar A.G., Flygare J., Gram M., Hansson S.R., Olsson M.L., Storry J.R., Allhorn M., Åkerström B. (2021). Human radical scavenger α1-microglobulin protects against hemolysis in vitro and α1-microglobulin knockout mice exhibit a macrocytic anemia phenotype. Free Radic. Biol. Med..

[24] Ahlstedt J., Tran T.A., Strand F., Holmqvist B., Strand S.-E., Gram M., Åkerström B. (2015). Biodistribution and pharmacokinetics of recombinant α1-microglobulin and its potential use in radioprotection of kidneys. Am. J. Nucl. Med. Mol. Imaging.

[25] Roth D., Gustafsson J., Warfvinge C.F., Sundlöv A., Åkesson A., Tennvall J., Gleisner K.S. (2022). Dosimetric quantities in neuroendocrine tumors over treatment cycles with 177Lu-DOTATATE. J. Nucl. Med..

[26] Kristiansson A., Örbom A., Vilhelmsson Timmermand O., Ahlstedt J., Strand S.-E., Åkerström B. (2021). Kidney Protection with the Radical Scavenger α1-Microglobulin (A1M) during Peptide Receptor Radionuclide and Radioligand Therapy. Antioxidants.

[27] Åkerström B., Gram M. (2014). A1M, an extravascular tissue cleaning and housekeeping protein. Free Radic. Biol. Med..

[28] Brieau B., Hentic O., Lebtahi R., Palazzo M., Ben Reguiga M., Rebours V., Maire F., Hammel P., Ruszniewski P., Fenaux P. (2016). High risk of myelodysplastic syndrome and acute myeloid leukemia after 177Lu-octreotate PRRT in NET patients heavily pretreated with alkylating chemotherapy. Endocr.-Relat. Cancer.

[29] Bodei L., Kidd M., Paganelli G., Grana C.M., Drozdov I., Cremonesi M., Lepensky C., Kwekkeboom D.J., Baum R.P., Krenning E.P. (2015). Long-term tolerability of PRRT in 807 patients with neuroendocrine tumours: The value and limitations of clinical factors. Eur. J. Nucl. Med. Mol. Imaging.

[30] Minczeles N.S., de Herder W.W., Konijnenberg M.W., Feelders R.A., Brabander T., Hofland J. (2022). Dose-Limiting Bone Marrow Toxicities After Peptide Receptor Radionuclide Therapy Are More Prevalent in Women Than in Men. Clin. Nucl. Med..

[31] Trivigno D., Bornes L., Huber S.M., Rudner J. (2013). Regulation of protein translation initiation in response to ionizing radiation. Radiat. Oncol..

[32] Sjögren S.E., Chen J., Mattebo A., Alattar A.G., Karlsson H., Siva K., Soneji S., Tedgård U., Chen J.J., Gram M. (2022). Targeting elevated heme levels to treat a mouse model for Diamond-Blackfan Anemia. Exp. Hematol..

[33] Olsson M.G., Nilsson E.J.C., Rutardóttir S., Paczesny J., Pallon J., Åkerström B. (2010). Bystander Cell Death and Stress Response is Inhibited by the Radical Scavenger α1-Microglobulin in Irradiated Cell Cultures. Radiat. Res..

[34] Gómez-Sámano M., Grajales-Gómez M., Zuarth-Vázquez J.M., Navarro-Flores M.F., Martínez-Saavedra M., Juárez-León Ó.A., Morales-García M.G., Enríquez-Estrada V.M., Gómez-Pérez F.J., Cuevas-Ramos D. (2017). Fibroblast growth factor 21 and its novel association with oxidative stress. Redox Biol..

[35] Lin Z., Zhou Z., Liu Y., Gong Q., Yan X., Xiao J., Wang X., Lin S., Feng W., Li X. (2011). Circulating FGF21 Levels Are Progressively Increased from the Early to End Stages of Chronic Kidney Diseases and Are Associated with Renal Function in Chinese. PLoS ONE.

[36] Olsson M.G., Rosenlof L.W., Kotarsky H., Olofsson T., Leanderson T., Morgelin M., Fellman V., Åkerström B. (2013). The radical-binding lipocalin A1M binds to a Complex I subunit and protects mitochondrial structure and function. Antioxid. Redox Signal..

[37] Kristiansson A., Davidsson S., Johansson M.E., Piel S., Elmér E., Hansson M.J., Åkerström B., Gram M. (2020). α1-Microglobulin (A1M) protects human proximal tubule epithelial cells from heme-induced damage in vitro. Int. J. Mol. Sci..

[38] Walrand S., Jamar F. (2021). Renal and Red Marrow Dosimetry in Peptide Receptor Radionuclide Therapy: 20 Years of History and Ahead. Int. J. Mol. Sci..

[39] Weiss R., Meersch M., Wempe C., von Groote T., Agervald T., Zarbock A. (2023). Recombinant Alpha-1-Microglobulin (RMC-035) to Prevent Acute Kidney Injury in Cardiac Surgery Patients: Phase 1b Evaluation of Safety and Pharmacokinetics. Kidney Int. Rep..

