# Supplementary material for: Recombinant α1-Microglobulin (rA1M) Protects against Hematopoietic and Renal Toxicity, Alone and in Combination with Amino Acids, in a 177Lu-DOTATATE Mouse Radiation Model
Source: Biomolecules. 2023 Jun 1;13(6):928. doi: 10.3390/biom13060928 (PMC10296637; doi:10.3390/biom13060928)
Supplement: Supplementary file 1 [file biomolecules-13-00928-s001.zip › biomolecules-2382361-supplementary.pdf]

Supplementary Material

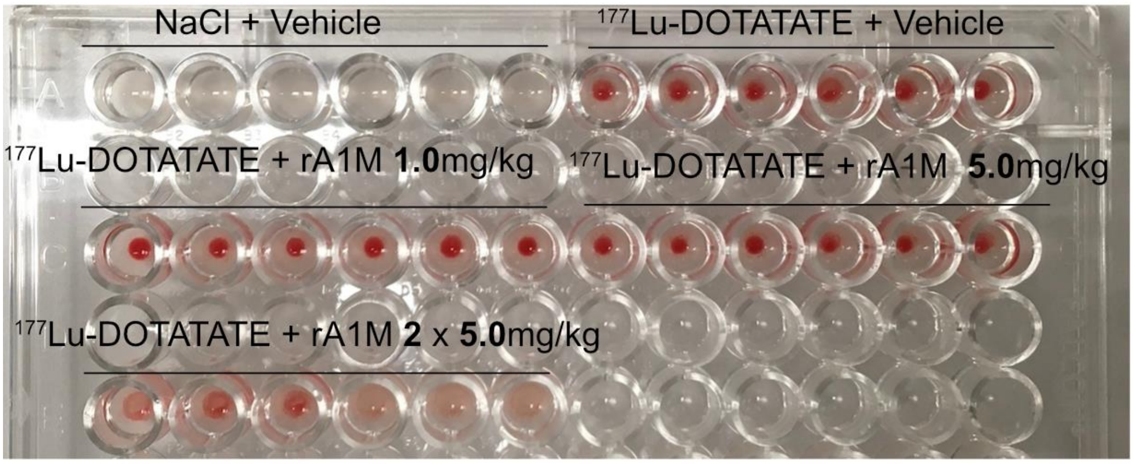

Supplementary Figure S1. Preservation of white marrow. Representative image of bone marrow isolated single cell suspensions. Image shows red pellet as an effect of the irradiation treatment on depletion of white marrow 4 days after dosing in animals from the different groups: NaCl and vehicle (volume corresponding to that of 150 MBq ^{177}Lu-DOTATATE or one dose of rA1M injection), 150 MBq, ^{177}Lu-DOTATATE with vehicle (volume corresponding to that of one dose of rA1M injection), or with rA1M (1, 5 or 2 x 5 mg/kg).

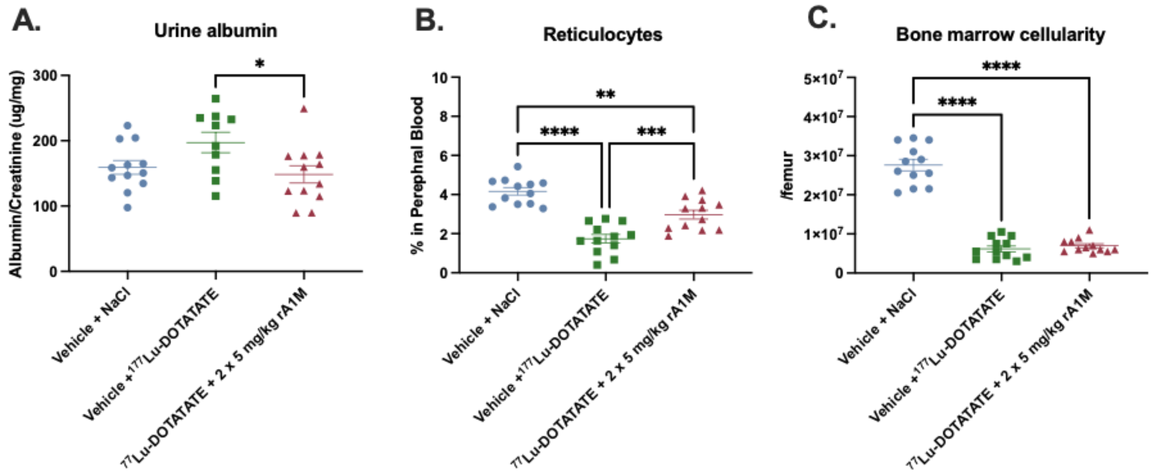

Supplementary Figure S2. The protective effects of rA1M. Urine albumin levels, corrected for creatinine (A), circulatory reticulocytes (B) and bone marrow cellularity (C) was measured 4 days after dosing in animals receiving NaCl and vehicle (volume corresponding to that of 150 MBq ^{177}Lu-DOTATATE respectively of rA1M injection), 150 MBq ^{177}Lu-DOTATATE with vehicle (volume corresponding to that of rA1M injection), or ^{177}Lu-DOTATATE with rA1M (2 x 5 mg/kg). Data is presented as scatter plots with mean ± SEM. Statistical comparison between groups was made with one-way ANOVA with a Šídák's multiple comparisons post hoc test. Only significant differences are presented in the figure. *$*P < 0.05$, ** $P < 0.01$, *** $P < 0.001$, **** $P < 0.0001$.*

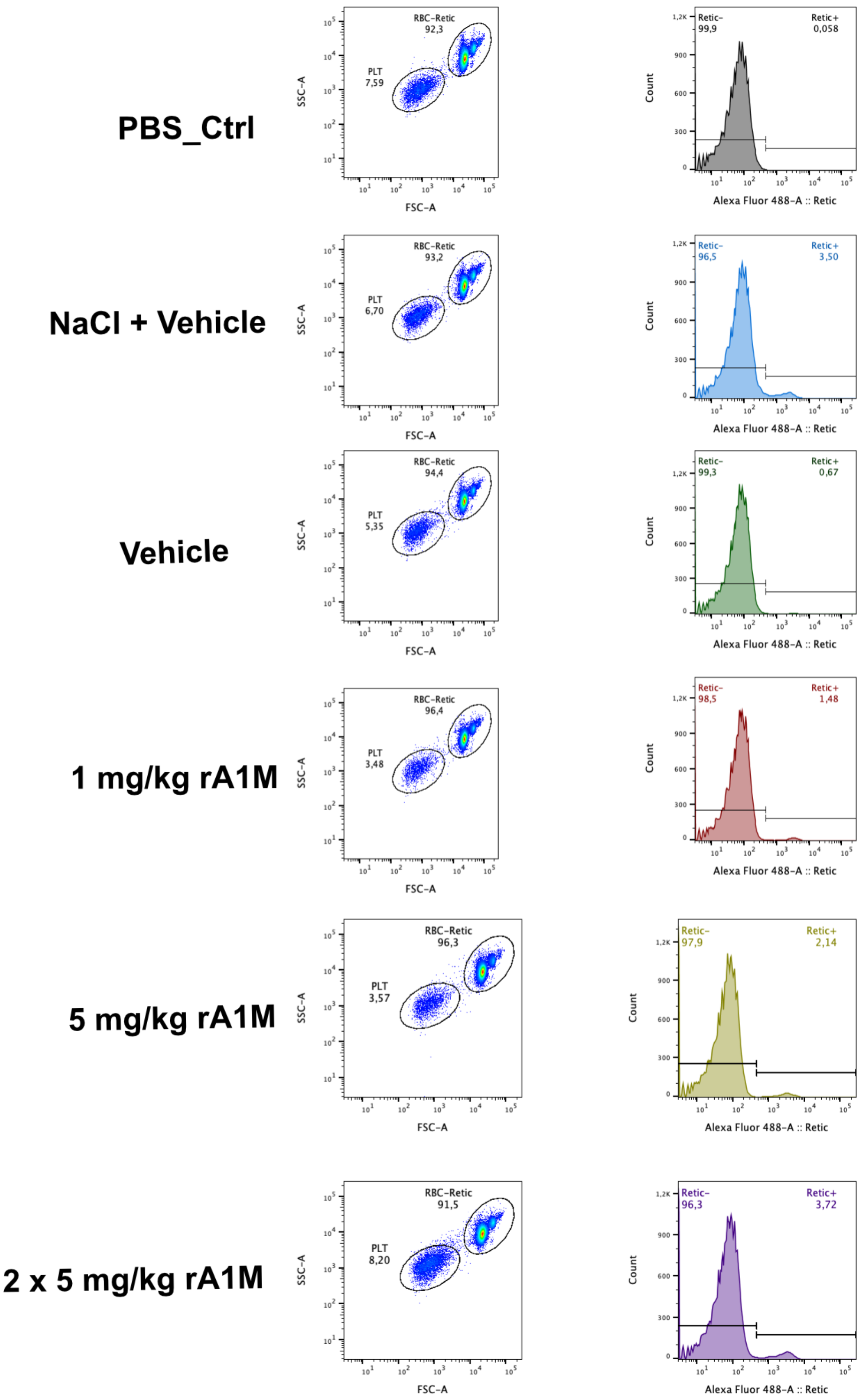

Supplementary Figure S3. The representative gating strategies for flowcytometric evaluation. In peripheral blood, reticulocyte percentage was determined using Retic-Count reagent. Cells were gated on RBCs-Retic population and the percentage of reticulocyte gating is set based on the PBS treated sample.

Supplementary Table S1. rA1M biodistribution with or without Vamin. Mean (%IA) and standard deviation from 10- and 60-minutes post-injections in kidney, liver, spleen, and femur (N = 4).

Group	*Organ*	*Time (min)*	*Mean (%IA)*	*SD*
rA1M	Kidney	10	0,766	0,067
rA1M	Liver	10	0,077	0,012
rA1M	Spleen	10	0,005	0,001
rA1M	Femur	10	0,009	0,001
rA1M + Vamin	Kidney	10	0,707	0,131
rA1M + Vamin	Liver	10	0,098	0,023
rA1M + Vamin	Spleen	10	0,005	0,001
rA1M + Vamin	Femur	10	0,009	0,001
rA1M	Kidney	60	0,155	0,060
rA1M	Liver	60	0,023	0,004
rA1M	Spleen	60	0,009	0,002
rA1M	Femur	60	0,021	0,002
rA1M + Vamin	Kidney	60	0,186	0,037
rA1M + Vamin	Liver	60	0,022	0,003
rA1M + Vamin	Spleen	60	0,007	0,002
rA1M + Vamin	Femur	60	0,018	0,003
